# MicroRNA-21 Contributes to Acute Liver Injury in LPS-Induced Sepsis Mice by Inhibiting PPAR*α* Expression

**DOI:** 10.1155/2020/6633022

**Published:** 2020-12-22

**Authors:** Xianjin Du, Miao Wu, Dan Tian, Jianlin Zhou, Lu Wang, Liying Zhan

**Affiliations:** ^1^Department of Critical Care Medicine, Renmin Hospital of Wuhan University, 238 Jiefang Road, Wuchang, Wuhan, Hubei 430060, China; ^2^Department of Emergency, Renmin Hospital of Wuhan University, 238 Jiefang Road, Wuchang, Wuhan, Hubei 430060, China; ^3^Department of Orthopedics, Renmin Hospital of Wuhan University, 238 Jiefang Road, Wuchang, Wuhan, Hubei 430060, China

## Abstract

The severity of sepsis may be associated with excessive inflammation, thus leading to acute liver injury. MicroRNA-21 is highly expressed in the liver of a variety of inflammation-related diseases, and PPAR*α* is also proved to participate in regulating inflammation. In the present study, the LPS-induced sepsis model was established. We found that microRNA-21 expression was upregulated in the liver of sepsis mice, and microRNA-21 inhibition significantly reduced the liver injury. The expression of liver injury markers, inflammation cytokines, and PPAR*α* in the septic mice was higher than in antagomir-21 treated septic mice. In addition, we also found that PPAR*α* is the target gene of microRNA-21; PPAR*α* antagonist GW6471 could reverse the effect of antagomir-21. In conclusion, our study illustrated that microRNA-21 exacerbate acute liver injury in sepsis mice by inhibiting PPAR*α* expression.

## 1. Introduction

Sepsis is a common cause of death in intensive care units [1]. It is a public health problem worldwide, and approximately 19 million people suffer from sepsis yearly [2]. Sepsis was defined as a “life-threatening organ dysfunction caused by a deregulated host response to infection” at the Sepsis-3 conference [3]. The acute liver injury occurs at any stage of sepsis; the dysregulation of hepatocyte function may be related to cytokine storm [4]. Liver injury can not only aggravate the development of the disease but also lead to death [5]. Despite the urgent need for effective therapeutic options, many new therapies have not improved the survival rate [6]. Therefore, understanding the pathogenesis of sepsis is very important for the treatment of sepsis.

Peroxisome proliferator-activated receptors (PPARs) are ligand-activated transcription factors belonging to a nuclear hormone receptor superfamily [7]. PPAR*α* is an isoform of peroxisome proliferator-activated receptors, which regulates adipocyte differentiation, fatty acid oxidation, and glucose metabolism [8]. More recently, emerging evidence revealed that PPAR*α* activation could reduce the inflammatory response by promoting NF-*κ*B inactivation [9]. In addition, the liver PPAR*α* expression was found to be disturbed during sepsis. In a murine model of sepsis, liver PPAR*α* expression was significantly associated with survival [10].

MicroRNA is a class of noncoding RNA, 19-22 nucleotides in length [11], which regulates gene expression at the posttranscriptional level by degrading message RNA or inhibiting its transcription [12]. MicroRNA microarray analysis showed the upregulation of microRNA-21 in sepsis patients [13]. Nevertheless, the role of microRNA-21 in sepsis-induced liver injury has not been fully elucidated.

In this study, we aim to explore the role of miR-21 and PPAR*α* in the pathogenesis of sepsis-induced liver injury. For this purpose, we used antagomir-21 to inhibit miR-21 expression in murine sepsis model and detected the levels of proinflammatory cytokines (TNF-*α*, IL-1*β*, and IL-6), liver injury markers (AST, ALT), and PPAR*α* expression.

## 2. Materials and Methods

### 2.1. Animal Models

All male C57BL/6 mice were obtained from Hubei Provincial Center for Disease Control and Prevention. The mice were housed in a pathogen-free facility with standard laboratory diet and water. At the age of 8 weeks, the mice were received three retroorbital intravenous injections of antagomir-21 (5'-UCAACAUCAGUCUGAUAAGCUA-3'; 16 mg/kg; *n* = 6), antagomir control (5'-AAGGCAAGCUGACCCUGAAGUU-3'; 16 mg/kg; *n* = 6), antagomir-21+GW6471 (16 mg/kg; 30 mg/kg; *n* = 6), phosphate saline (PBS; *n* = 12). Six PBS-treated mice were used as controls; the other mice received intraperitoneal injection of 5 mg/kg lipopolysaccharide to induce sepsis. At 24 h after LPS injection, all mice were sacrificed.

All experiments were performed in accordance with the *Guidelines for the Care and Use of Laboratory Animals* published by the National Institutes of Health (2011).

### 2.2. Cell Culture

Mouse Biliary Duct Epithelial Cells and 293T cells were obtained from Newgainbio (Wuxi, China) and cultured in DM/F12 medium or Dulbecco's Modified Eagle's Medium (DMEM) supplemented with 10% fetal bovine serum (FBS), 100 U/mL penicillin and 100 *μ*g/mL streptomycin under 5% CO2 at 37°C.

### 2.3. Luciferase Reporter Assay

MiR-21 mimics (5'-UAGCUUAUCAGACUGAUGUUGA-3') and NC-mimics (5'-UUCUCCGAACGUGUCACGUTT-3') were purchased from Ribobio (Guangzhou, China). The pGL3 Luciferase Reporter Vectors (Promega) containing the PPAR*α*-MT (5'-AAAAAAUCUGUUAGAUAAGCUA-3') and PPAR*α*-MuT (5'-AAUUAUAGUCAUACUAUUCGAA-3') sequences were cotransfected with miR-21 mimics or NC-mimics (50 nM) into 293T and MBDEC cells. All transfections were performed using Lipofectamine 3000 (Invitrogen, USA). After 24 hours of incubation, 1×PLB was used to lyse the cells, and the luciferase activities were measured using the Dual-Luciferase Reporter Assay System (Promega).

### 2.4. RNA Isolation and qRT-PCR

Total RNA was extracted from snap frozen mouse liver samples using TRIzol reagent (Invitrogen, USA). MiR-21 and mRNA levels were quantified by qRT-PCR assay. For miRNA, U6 was applied as endogenous control. For mRNA, GAPDH was used as endogenous control. All reactions were run on the ABI 7500Real-Time PCR System (Life Technologies, USA). The relative expression was calculated using 2^-*ΔΔ*CT^ method. All primers used in this study are as follows.

MiR-21 (stem-loop RT primer): 5'-GTCGTATCCAGTGCAGGGTCCGAGGTATTCGCACTGGATACGACTCAACA-3'

MiR-21-F: 5'-GTGCAGGGTCCGAGGT-3'

MiR-21-R: 5'-GCCGCTAGCTTATCAGACTGATGT-3'

U6-F: 5'-AGCCCGCACTCAGAACATC-3'

U6-R: R: 5'-GCCACCAAGACAATCATCC-3'

GAPDH-F: 5'-CGTCCCGTAGACAAA ATGGTGAA-3'

GAPDH-R: 5'-GCCGTGAGTGGAGTCATACTGGAA CA-3'

PPAR*α*-F: 5'-AACCTGAGGAAGCCGTTCTGTGACAT-3'

PPAR*α*-R: 5'-GACCAGCTGCCGAAGGTCCACCAT-3'.

### 2.5. ELISA

The TNF-*α*, IL-1*β*, and IL-6 levels in liver or cultured supernatant were quantified using the Mouse TNF-*α* Precoated Elisa kit, Mouse IL-1*β* Precoated Elisa kit, and Mouse IL-6 Precoated ELISA kit, respectively.

### 2.6. Metabolic Analyses

Serum alanine aminotransferase (ALT) and aspartate aminotransferase (AST) levels were determined using the VITROS350 chemistry system (Johnson & Johnson, USA).

### 2.7. Histological Analysis of Liver

Liver samples from mice were fixed in 4% paraformaldehyde, then stained with hematoxylin and eosin. The liver damage photos were observed and recorded under light microscopy.

### 2.8. Western Blot

The nuclear protein of liver samples was extracted using NE-PER™ Nuclear Extraction Reagents (Thermo Fisher, USA). Protein was quantified using BCA Protein Assay (Thermo Fisher, USA). Total protein (50 *μ*g) was separated by sodium dodecyl sulphate-polyacrylamide gel electrophoresis (SDS-PAGE) and transferred to polyvinylidene fluoride (PVDF) membranes (Millipore, USA). Membranes were incubated with the primary antibodies (1 : 1000) overnight at 4°C. After HRP-conjugated secondary antibodies (1 : 5000) incubation; the protein bands were visualized using the Clarity Western ECL Substrate (Bio-Rad, USA).

### 2.9. Statistical Analyses

Student's *t* test and one-way analysis of variance were used to analyze the significance between groups. *p* < 0.05 was considered statistically significant. Data analysis was performed using SPSS 22.0 (IBM, Chicago, USA), and figures were designed using GraphPad Prism 8.3.0.

## 3. Results

### 3.1. Expression of miR-21 Is Increased in the Liver of Sepsis Mice

**Figure 1:**
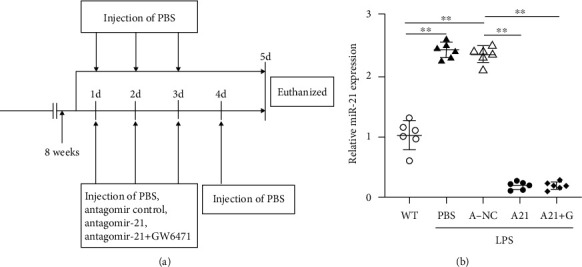
MiR-21 expression was increased in the liver of sepsis mice. (a) Mouse study design. (b) The miR-21 expression in the liver of the wide-type mice treated with PBS (WT) and the LPS-induced mice treated with PBS, antagomir-21 control (NC), antagomir-21 (A21), antagomir-21, and PPAR*α* antagonist GW6471 (A21+G). QRT-PCR assay was used to detect the expression of miR-21. ∗∗*p* < 0.01.

The animal experiments were performed according to the design (Figure 1(a)). After these mice were sacrificed, we collected the serum of the mice and isolated the livers. Subsequently, the expression of miR-21 in the liver was detected using qRT-PCR (Figure 1(b)). The results showed that miR-21 expression was significantly upregulated in septic mice; nevertheless, the injection of antagomir-21 totally blocked the upregulation of miR-21, suggesting that the sepsis mouse models with miR-21 inhibition were successfully established.

### 3.2. Antagomir-21 Reduces Liver Injury and Inflammation in Sepsis Mice

**Figure 2:**
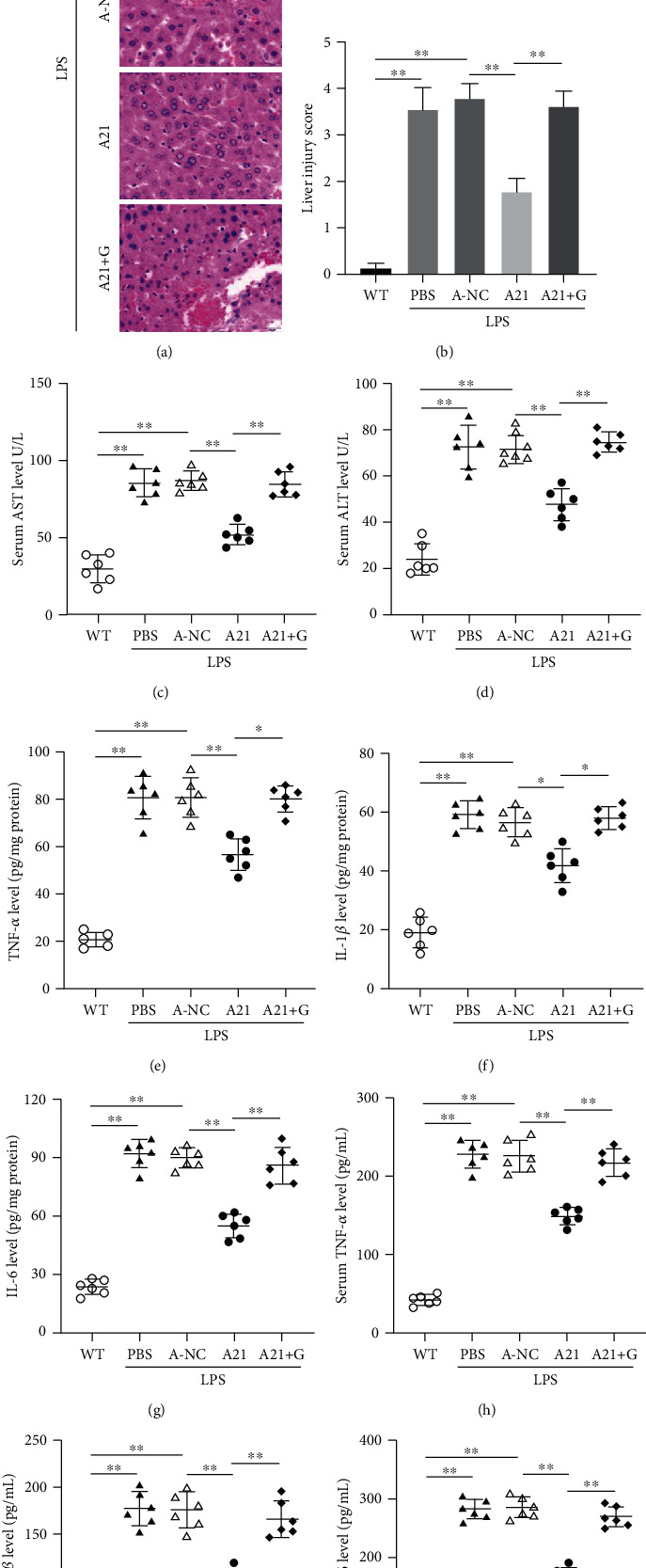
Antagomir-21 reduces liver injury and inflammation. (a, b) Histological analysis of livers. Tissue damages were scored in a scale of 0–4, with 0, 1, 2, 3, and 4 corresponding to 0%, <25%, 26%–50%, 51%–75%, and ≥76% of liver injury, respectively. (c, d) The serum aspartate aminotransferase (AST) and alanine aminotransferase (ALT) levels. (e–g) ELISA assay was used to determine the levels of TNF-*α*, IL-1*β*, and IL-6 in livers. (h–i) ELISA assay was used to determine the levels of TNF-*α*, IL-1*β*, and IL-6 in serum. ∗*p* < 0.05, ∗∗*p* < 0.01.

To further investigate the function of miR-21 in sepsis-induced liver injury, we next evaluated the pathological damage of livers and the levels of proinflammatory cytokines. As shown in Figures 2(a) and 2(b), the degree of liver damage in septic mice was significantly increased. Antagomir-21 administration alleviated the liver injury; however, in the antagomir-21 and GW6471 coadministration group, the degree of liver injury showed no changes. As expected in these mice of sepsis, the expression of liver injury markers (serum AST and serum ALT; Figures 2(c) and 2(d)) and proinflammatory cytokines (TNF-*α*, IL-1*β*, and IL-6; Figures 2(e)–2(j)) was notably reduced by antagomir-21. Nevertheless, GW6471 restored the levels of these cytokines again. To sum up above results, it demonstrated that miR-21 inhibition strongly decreased the liver injury and inflammation in septic mice. But the effects produced by miR-21 inhibition were reversed by PPAR*α* antagonist GW6471.

### 3.3. MiR-21 Directly Interacts with PPAR*α*

**Figure 3:**
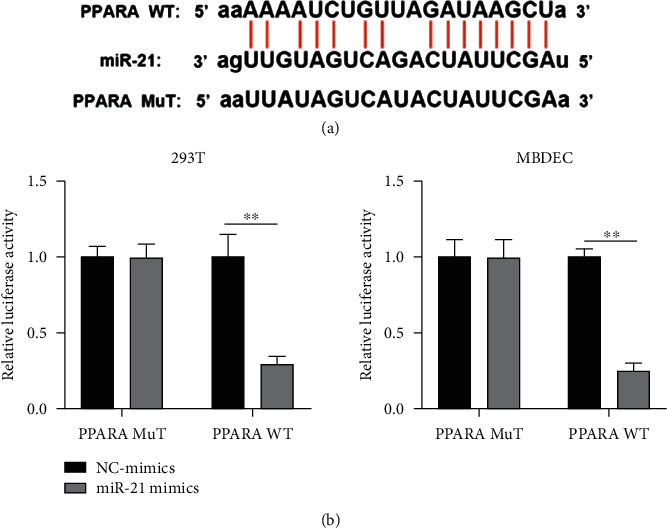
MiR-21 directly interacts with PPAR*α*. (a) The putative binding sites between PPARA WT and miR-21. (b) Luciferase activity was detected in luciferase reporter vectors harboring PPARA WT/MuT sequences and miR-21 mimics cotransfected 293T and MBDEC cells. ∗∗*p* < 0.01.

It is well known that miRNAs participate in a variety of physiological activities by regulating gene expression at the posttranscriptional level. To investigate the molecular mechanisms of miR-21 in sepsis, we used TargetScan to identify the potential genes, and PPAR*α* was found to be one of the most relevant genes to sepsis. Starbase (http://starbase.sysu.edu.cn) was used to predict the putative binding sites between PPAR*α* and miR-21 (Figure 3(a)). Luciferase report assay was performed to test whether miR-21 can directly bind to PPAR*α*. As shown in Figure 3(b), in the 293T and MBDEC cells that cotransfected with PPAR*α* WT and miR-21 mimics, the luciferase activities were significantly reduced, but there were no changes in the PPAR*α*-MuT group. The results indicated that PPAR*α* is a target gene of miR-21.

### 3.4. Antagomir-21 Reduces Liver Injury and Inflammation by Restoring PPAR*α* Expression

**Figure 4:**
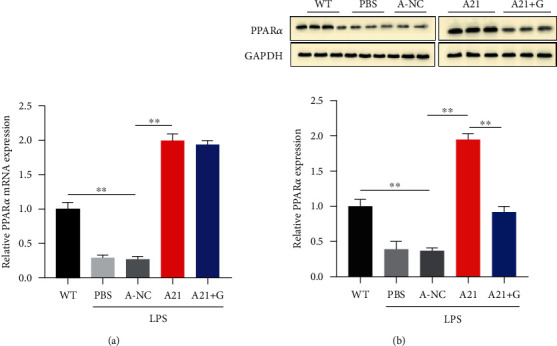
Antagomir-21 reduced liver injury and inflammation by restoring PPAR*α* expression. (a) The expression of PPAR*α* mRNA in livers was detected by qRT-PCR. (b) The expression of PPAR*α* nuclear protein in livers was detected using western blot. ∗∗*p* < 0.01.

To further verify whether miR-21 regulates the expression of PPAR*α* in the liver. We detected the PPAR*α* expression in the liver of five groups; three liver samples was randomly selected from each groups (Figures 4(a) and 4(b)). The total RNA was extracted from liver tissues, and the protein was extracted from the nucleus. The results showed that PPAR*α* expression in PBS and AC groups was markedly decreased compared with the WT group. In addition, antagomir-21 treatment increased the PPAR*α* expression in the liver, but GW6471 inhibited the level of PPAR*α* nuclear protein. Aggregating all the results, we found that antagomir-21 alleviated liver injury and inflammation by restoring PPAR*α* expression.

## 4. Discussion

This study illustrated that miR-21 suppression attenuated liver injury in LPS-induced sepsis mice, by potentiating PPAR*α* expression, which suggested a contribution of miR-21 in the pathogenesis of sepsis-induced liver injury. In addition, antagomiR-21 and PPAR*α* represented anti-inflammatory activities in septic mice. These findings demonstrated that the miR-21/PPAR*α* pathway might serve as a potential target in sepsis therapy.

A proinflammatory status is the key feature of sepsis, and the liver plays an important role in inflammation [14]. According to reports, miR-21 is upregulated in various inflammatory diseases, including myocardial injury [15], nonalcoholic steatohepatitis [16], and osteoarthritis [17]. However, the role of miR-21 in sepsis has not been fully elucidated. At first, we confirmed that miR-21 expression was elevated in the liver of LPS-induced sepsis mice. Then, to investigate the molecular mechanism of miR-21 in sepsis, we screened out some genes with miR-21 binding sites. Previous reports have shown that PPAR*α* expression is reduced in the liver of NASH patients, and PPAR*α* activation inhibits liver fibrosis in mice [18, 19]. These researches promoted us to verify the connection between miR-21 and PPAR*α*. Subsequently, we found that in the liver of sepsis mice model, PPAR*α* expression was decreased when miR-21 expression was increased. In addition, antagomir-21 restored PPAR*α* expression and attenuated sepsis-induced liver injury, whereas PPAR*α* antagonist GW6471 blocked the inhibitory effect of antagomir-21 on liver injury. Altogether, our study indicated that miR-21 regulated liver inflammation through PPAR*α* inhibition.

Nevertheless, there are several limitations in our study. Recent studies reported that miR-21 was primarily expressed in biliary and inflammatory cells in the liver, rather than in hepatocytes. In this study, the cellular source of miR-21 was not elucidated; the function of miR-21/PPAR*α* axis needs to be further explored.

In addition, miR-21 and PPAR agonists are considered druggable targets [20, 21]. This study showed that the mir-21/PPAR*α* pathway might be an interesting new strategy for sepsis treatment. Some PPAR agonists, however, show side effects that cause them to be discontinued [22], and mir-21 antagonism treatment may also have side effects. Therefore, rigorous study design and safety monitoring are essential.

In conclusion, in LPS-induced sepsis mice model, we demonstrated that miR-21 contributed sepsis-induced liver injury and inflammation by inhibiting PPAR*α* expression. Therefore, this study may provide an attractive potential target for the treatment of sepsis.

## Data Availability

The data that support the findings of this study are available from the corresponding author upon reasonable request.
